# Understanding Town Centre Performance in Wales: Using GIS to Develop a Tool for Benchmarking

**DOI:** 10.1007/s12061-021-09417-z

**Published:** 2021-08-20

**Authors:** Samuel Jones, Andy Newing, Scott Orford

**Affiliations:** 1 Wales Institute of Social and Economic Research and Data (WISERD), School of Social Sciences, Cardiff University, 38 Park Place, Cardiff, CF10 3BB UK; 2 School of Geography, University of Leeds, Leeds, LS2 9JT UK; 3 Wales Institute of Social and Economic Research and Data (WISERD), School of Geography and Planning, Cardiff University, Glamorgan Building, King Edward VII Avenue, Cardiff, CF10 3WA UK

**Keywords:** Town centres, Typology, Spatial interaction modelling, k-Means clustering, Benchmarking, Wales

## Abstract

Welsh Government policy establishes town centres as central places of community activity and local prosperity, recognising the positive impact towns have on the local economy and the well-being and cohesion felt amongst local communities. In light of this, recent declines in the usage of town centres are a major cause for concern. These have not been experienced uniformly across all towns, with some towns out-performing others. This paper applies principles outlined in Welsh Government’s Planning Policy Wales to develop a tool which classifies a sample of 71 towns and cities in Wales based on their centre and catchment characteristics. Catchment areas have been delineated using a Spatial Interaction Model to account for complex consumer behaviours and competition between centres. The tool identifies six distinct types of towns alongside key socio-economic catchment area characteristics. Once developed, we demonstrate our tool’s application by exploring variations in town centre performance between and within each town type. Case study examples exemplify how policymakers may use this tool to benchmark between towns, evaluating the suitability of a town’s retail offering based on its performance relative to the benchmark, guiding decisions relating to the types of businesses and uses a town should pursue to improve its appeal to its catchment community. In conclusion, several recommendations to policymakers are suggested.

## Introduction

A town centre’s purpose, at its most basic, is to meet the needs of those who use it (BIS, 2011; Coca-Stefaniak, 2013). Over time, the range of uses of towns has diversified making them complex, multi-dimensional and difficult to define (Coca-Stefaniak, 2013; Astbury & Thurstain-Goodwin, 2014; Millington, Ntounis, Parker & Quin, 2015). The Department for Business, Innovation and Skills’ (BIS) definition states that towns must offer access to retail, service and leisure businesses; offer opportunities for employment; offer good transport links and; be recognised as a town centre by the local community (Coca-Stefaniak, 2013). It is these latter characteristics that distinguish town centres from other purpose-built retail centres, where non-retail related employment, transportation and social cohesion are less of a focus.

Town centres, therefore, meet a different set of needs within local communities to purpose-built centres, influencing economic performance, social cohesion and wellbeing at local and regional levels (BIS, 2011; Coca-Stefaniak, 2013; Welsh Government, 2018, 2019). Their importance has been recognised by Welsh Government’s Planning Policy Wales (PPW) where settlements are identified as ‘the heart of communities’ and ‘central to community activity and local prosperity’ (pp. 46 and pp. 63 respectively, Welsh Government, 2018).

Changing consumer trends, with consumers shopping in out-of-town locations or via other mediums, such as online, have led to a recent decline in the use of town centres. The resulting detrimental impact on local communities has caused concern. In an attempt to reverse these trends government policies have focused on improving the vitality, viability and resilience of towns with varying degrees of success (Welsh Government, 2014a; Wrigley & Dolega, 2011; Wrigley & Lambiri, 2014), largely depending on the characteristics of a town centre or on its geographic location (Cox, Thurstain-Goodwin & Tomalin, 2000; Dolega & Lord, 2020). However, differences in performance are often observed between town centres of similar characteristics and, as Wrigley and Lambiri point out, “Understanding… what makes [a town centre or high street] perform relatively better or worse than its peers remains a key question” (Wrigley & Lambiri, 2014). This study seeks to address this question in a Wales context by applying Welsh Government’s recently updated planning policies through the development and application of a new tool.

Within PPW, Welsh Government highlights the importance of using robust evidence to support strategies intended to improve town centre performance (Paragraph 4.3.6, Welsh Government, 2018). As part of this, they suggest that stakeholders would benefit from gaining a better understanding of both town characteristics and the characteristics of town catchment areas, citing the importance of the development of a typology of towns in Wales (Paragraph 4.3.12, Welsh Government, 2018; Paragraph 4.3.13, Welsh Government, 2018).

As a result, the research presented here is split into two interlinked aims:

Firstly, we develop a tool which consists of a typology, based on the town characteristics of 71 of Wales’ largest towns and cities, and a dataset of catchment characteristics, derived via the delineation of catchment area boundaries.

Secondly, we demonstrate the application of this tool, showing its potential to improve stakeholders’ understanding of town centre performance differentials.

By taking this approach we hope to provide the robust, data-driven evidence cited by PPW as being an important basis for policy decisions. In the following sections we report on the creation of our tool from both a theoretical and practical perspective. We begin by reviewing the relevant literature before outlining the modelling approach used to construct bespoke catchment areas. Subsequently we step-through the creation of our powerful typology of Welsh town centres and illustrate its application, alongside our derived catchment area characteristics, to town centre performance management. We conclude with recommendations for the use of this tool by stakeholders in Wales, with wider implications for broader application in other national contexts.

## Literature

Town centres are, by definition, distinctly different in character and remit to purpose-built shopping spaces. Their multi-dimensional nature makes them important locations for economic, civic and social functions within local communities, providing access to employment opportunities, retail, services, cultural activity and transportation (Coca-Stefaniak, 2013; Millington et al., 2015; Grimsey, 2018; Welsh Government, 2019). Historically they were places key to establishing feelings of cohesion and belonging amongst local communities (Findlay & Sparks, 2012; Birkin, Clarke & Clarke, 2017), feelings that Welsh Government’s recent Planning Policy Wales (PPW) is keen to establish and strengthen through its “town centres first” policy (Welsh Government, 2018). PPW also establishes towns as the most sustainable places for new development and regeneration in Wales, underlining the importance of these places to Welsh Government achieving the sustainability goals highlighted in the Well-being of Future Generations (Wales) Act (2015) (Welsh Government, 2015, 2018, 2019). Recent declines in the usage of towns are, therefore, a cause for concern, and Welsh Government are keen to try to reverse these trends.

### The Importance of Adaptation in Reversing Trends of Decline

The reasons for the recent declines in town centre usage have been well documented. Prevailing economic conditions in the UK following the global financial crisis and the EU referendum, including a real-terms reduction in gross disposable household income and reduced consumer confidence, has led to a reduction in consumer spending, with town-based businesses suffering as a result (Coca-Stefaniak, 2013; Dolega & Lord, 2020; Wrigley & Dolega, 2011). Furthermore, profit margins amongst bricks-and-mortar retailers have been squeezed by rising inflation, wages, rental prices and business rates along with a less favourable exchange rate, leading to a 2.9% average increase in running costs (BIS, 2011; Powe, 2012; Grimsey, 2018). Recent evidence given to the UK Parliament’s Business, Energy and Industrial Strategy Committee suggests that the coronavirus lockdown will add to these difficulties, with independents and other small businesses suffering the most (The Guardian, 2020).

Despite this, retailing has become more important to the UK economy in recent years, accounting for 11% of GVA, 20.5% of employment and 15.5% of businesses in 2016 (Grimsey, 2018). Although the proportion of retail sales taking place online increased from 10.4% in 2013 to 17.9% in 2017, physical stores are still important in four out of five transactions (Dolega et al., 2019; Grimsey, 2018). Despite the increased contribution of retailing to the economy and the continued importance of physical stores many towns are experiencing decline, failing to keep up with changing consumer preferences (Coca-Stefaniak, 2013; Dolega et al., 2019; Jackson, 2006) and more recently as a result of changes in mobility, work and leisure related behaviours driven by coronavirus lockdown periods (McCulloch, 2021).

A shift towards online retail has caused many comparison retailers, once ubiquitous to town centres in the UK, to either close or to relocate to out-of-town retail parks (BIS, 2011; Wrigley & Lambiri, 2014; Singleton et al., 2016; Dolega & Lord, 2020). Retail parks are often more attractive to retailers and consumers alike, offering better economies of scale, modern fit-for-purpose retail stock, covered pedestrianised areas, convenient free parking and lower levels of congestion (Astbury & Thurstain-Goodwin, 2014; Powe, 2012). Town centres have struggled to compete, with a decline in businesses and footfall leading to many being termed ‘ghost towns’ (Dolega & Lord, 2020). Moreover, recent drives towards convenience culture, discounters, services, leisure and the experience economy have meant that traditional retailing can no longer be relied upon to make town centres vibrant and viable places (Hood, Clarke & Clarke, 2016; Birkin et al., 2017; Dolega et al., 2019; Dolega & Lord, 2020).

Adapting a town’s characteristics (the supply side) to these consumer trends (the demand side) (Astbury & Thurstain-Goodwin, 2014; Wrigley & Lambiri, 2014; LDC, 2017a) is key to improving a town’s resilience (Wrigley & Dolega, 2011; Findlay & Sparks, 2012; Millington et al., 2015; Grimsey, 2018; Dolega & Lord, 2020). Although the performance of a town centre is a result of the interaction between the demand and supply sides, from a policy perspective, policymakers can do little to influence demand at the local level but they can influence centre characteristics such as business rates, cost and availability of parking, as well as retail mix and the look and feel of the general retail environment.

### The Influence of Supply and Demand on Town Centres

#### Demand Characteristics

The majority of demand for retail services within a town centre comes from its catchment area (Wrigley & Lambiri, 2014; Dolega, Pavlis & Mingleton, 2016). Catchment areas are, however, complicated, with many consumers not visiting their local centre or patronising multiple centres as part of multi-purpose trips, making them difficult to delineate (Dolega et al., 2016). Spatial Interaction Models (SIMs) have been used frequently by the retail sector to predict the patronage of individual stores or retail centres, developing catchment areas in the process. Such models have also been applied to town centres in England to ascertain catchment characteristics (Wilson, 2010; Birkin et al., 2017; Dolega et al., 2016). Accessibility is also a key driver of centre patronage decisions, particularly in rural Wales where travel is often restricted by the topography, characterised by long journey times to major centres and restricted by limited access to public transport (WRO, 2007a; Dolega et al., 2016).

The demographic and socio-economic characteristics of those visiting town centres are often overlooked (Singleton et al., 2016). However, a potential patron’s characteristics often influences their expectations of a town centre and understanding these expectations is key to tailoring a town’s supply-side characteristics, making it more attractive (BIS, 2011; Wrigley & Lambiri, 2014).

Demographic characteristics such as age, social class, employment rates, health and ethnicity influence shopping habits, preferences and disposable income (BIS, 2011; Coca-Stefaniak, 2013; Parker, Ntounis & Quin, 2014; Wrigley & Lambiri, 2014; Singleton et al., 2016). Although towns situated amongst more affluent communities often perform better, studies have shown that centres situated in less affluent areas can perform well when attuned to the needs and characteristics of their catchment area population (Coca-Stefaniak, 2013; Wrigley & Dolega, 2011; Wrigley & Lambiri, 2014).

#### Supply Characteristics

Supply-side characteristics are fundamental to a centre’s ability to attract patrons (Singleton et al., 2016). Both large and small towns are often attractive to patrons, with large towns offering greater diversity of uses (Singleton et al., 2016; Wrigley & Dolega, 2011) and smaller towns benefitting from a reliable local catchment population (Wrigley & Lambiri, 2014), or USPs centred around independent retailers, culture or the natural environment (Wrigley & Dolega, 2011). Medium sized towns do not typically benefit from these characteristics, making them less attractive and more vulnerable (Singleton et al., 2016).

Towns with a diverse mixture of businesses are often more attractive, with large multiples, often more resilient to change, acting as ‘magnet’ (or ‘anchor’) which attract consumers and drive footfall (Wrigley & Dolega, 2011), and small independents giving a centre ‘personality’ (Wrigley & Dolega, 2011). Leisure and services which cannot be obtained online such as food, drink, beauty, health or education, as well as residential dwellings and major workplaces, are often good drivers of footfall throughout the day and evening (Coca-Stefaniak, 2013; Dolega & Lord, 2020; Grimsey, 2018; Powe, 2012; Wrigley & Lambiri, 2014).

When considering a town’s business mix, planners are encouraged to undertake a ‘Needs Test’ to ensure that a town’s characteristics align to the demands of its catchment (Welsh Government, 2018). The complexity of these relationships means that town centre management is becoming increasingly data driven and evidence based.

### Data Driven Decision Making in Town Centre Management

Town centre management is an important method of ensuring that a centre diversifies in line with consumer expectations (Wrigley & Lambiri, 2014). Within Wales, local decision making is framed by key national legislation and planning policies, including the National Development Framework (NDF), Planning Policy Wales (PPW) and the Well-being of Future Generations (Wales) Act 2015 (WFGA). These should be applied conjunctively, ensuring that the key national goals (NDF) are addressed by the planning system (PPW) in a way that aligns to Welsh Government’s sustainability principles (WFGA) (Welsh Government, 2015, 2018, 2019).

Regular meetings of local management groups provide stakeholders from both the public and private sector with opportunities to tailor national, regional and local government policies to their local town (Astbury & Thurstain-Goodwin, 2014; Jackson, 2006). These groups should be responsible for making key decisions about the retail mix, the physical environment, seasonal opening hours, or car parking policies (Astbury & Thurstain-Goodwin, 2014; Coca-Stefaniak, 2013; Powe, 2012; Welsh Government, 2014a, 2014b; Wrigley & Lambiri, 2014), ensuring that their town meets consumer expectations and maintains an advantage over its competitors, whilst also remaining within the remit of broader government policy (Wrigley & Dolega, 2011). Effective town centre management requires empirical evidence in the form of key performance indicators or metrics (Grimsey, 2018; Welsh Government, 2018), providing stakeholders with the information they need to understand a centre’s strengths and weaknesses, assess the effectiveness of management strategies and to guide investment (Cox et al., 2000; ODPM & CASA, 2001; Findlay & Sparks, 2012; Coca-Stefaniak, 2013).

### Metrics for Measuring Town Centre Performance

Metrics used to measure town centre performance should be temporally and spatially consistent and simple to understand, avoiding misinterpretation by nonexpert audiences (Coca-Stefaniak, 2013; Findlay & Sparks, 2008; Hogg et al., 2007). Unfortunately, since the cessation of the UK Census of Distribution in 1971, comprehensive and consistent metrics relevant to town centres have not been readily available in the UK (Findlay & Sparks, 2008). This makes the effectiveness of town centre management strategies difficult to evaluate (Dolega et al., 2019).

Stretched local authority budgets often prohibit them from collecting this information and data collected by private organisations, such as the Local Data Company (LDC), are relied upon to fill this gap (Astbury & Thurstain-Goodwin, 2014; Findlay & Sparks, 2008, 2012). However, data licenses can be prohibitively expensive (Wrigley & Lambiri, 2014) and, as these data are not collected for policy evaluation, they can be biased towards larger, commercially viable towns (Findlay & Sparks, 2008, 2012). Despite this, such data are commonly used as measures of town centre performance and metrics include proportions of vacant premises, footfall counts and visitor perceptions (Findlay & Sparks, 2010; BIS, 2011; Wrigley & Lambiri, 2014).

Retail vacancy rates, one of the most used metrics, are a good indicator of problems within towns (Dolega & Lord, 2020). LDC’s retail vacancy rates can be split into three types: (1) the total number of vacant retail premises, (2) churn, where premises are vacant for less than a year; and, (3) persistent vacancy, where premises are left vacant for over 3 years (LDC, 2017a). A small amount of churn is desirable, suggesting that a town is adapting to consumer trends (Findlay & Sparks, 2010; Wrigley & Lambiri, 2014), whereas high proportions of persistently vacant premises can indicate unattractive towns with poor quality retail stock that needs repurposing (Findlay & Sparks, 2010, 2012; Wrigley & Dolega, 2011). Despite the licensing restrictions, the national coverage and relatively consistent definitions of LDC’s vacancy rates data make them a useful metric for making spatial and temporal comparisons between towns in a process known as ‘benchmarking’ (Cox et al., 2000; Findlay & Sparks, 2012).

### The Importance of Typologies in Benchmarking

As towns are unique (BIS, 2011; Coca-Stefaniak, 2013) a ‘one size fits all’ approach to benchmarking is not appropriate (Cox et al., 2000), with many preferring to classify like-for-like centres into groups first, prior to making comparisons (Coca-Stefaniak, 2013; Dolega et al., 2019; Wrigley & Dolega, 2011).

Attempts to classify UK retail centres are not new and numerous classifications have been developed by government departments, private companies, or academic research (Dolega et al., 2019). Frameworks produced by government departments help planners classify their local towns based on their size, business mix, position in the retail hierarchy and catchment characteristics (BIS, 2011; Coca-Stefaniak, 2013). Commercial organisations, such as Experian, have taken this a step further, producing data-driven measures of a town’s vitality, resilience or economic outlook based on composite indices which are rarely available in the public domain (Dolega et al., 2019; Singleton et al., 2016). Alternatively, academic research has resulted in typologies which categorise centres hierarchically based on business structure, such as ranks of multiple comparison retailers (e.g. Hall et al., 2001; Reynolds & Schiller, 1992; Schiller & Jarrett, 1985 (from Dolega et al., 2019)), or measures of attractiveness (Dolega et al., 2016). Alternatively, classifications have been developed based on town characteristics beyond business structure, such as e-resilience (Singleton et al., 2016), footfall (Mumford, Parker, Ntounis & Dargan, 2017), vacancy rates (Dolega & Lord, 2020) or socio-economic characteristics (Coca-Stefaniak, 2013).

The number of typologies which focus on Wales is more limited. In 2011, Woods used demographic, social and economic characteristics, along with a town’s service function, to identify six town types (Woods, 2011). More recently, research funded by the Carnegie Trust UK produced the Understanding Welsh Places web-tool (UWP, 2021), which contains two seven cluster classifications of towns with a population of 2000 people or more. The first, based on the demographic and socio-economic characteristics of a town’s contiguous built-up area (CBUA) and the second describing a town’s relationship with surrounding towns based on its public, commercial and social economy assets (UWP, 2021).

Additionally, Dolega et al. (2019) have produced a typology for all ‘shopping spaces’ in Great Britain that utilises a combination of the mix of commercial businesses as well as a space’s size and economic health. Although their typology does include 122 places in Wales, the influence of English towns on its construction does leave a gap for an alternative Wales-specific typology more attuned to the nuanced characteristics and performance trajectories of town and city centres in Wales.

This research seeks to fill this gap by developing a tool, consisting of a Wales-specific typology of towns that can be used in conjunction with their catchment characteristics to provide stakeholders with the evidence required to benchmark one Welsh town against another.

## Developing Our Tool for Benchmarking Towns in Wales

This section covers the development of the elements of our new tool for benchmarking towns in Wales. A description of the data used is followed by sections briefly detailing the approaches used to delineate our town centre catchments and to develop our town centre typology.

### Data

Data for this analysis were collected as part of the LDC’s rolling survey of > 3000 shopping spaces across Great Britain (LDC, 2018). Each location is visited at least once per year, with data for larger locations updated bi-annually. Towns are defined using the Department for Communities and Local Government (DCLG) town centre boundaries which was the definition used by the LDC for releasing their data (Thurstain-Goodwin & Unwin, 2000; Wrigley & Dolega, 2011; Dolega et al., 2016). Other boundaries have subsequently been made available, such as those released by the ESRC Consumer Data Research Centre and will be considered in future research (Pavlis et al., 2018). Data on business types are categorised into multiple, independent, comparison, convenience, leisure, services and miscellaneous (Dolega et al., 2016; LDC, 2017a). Data on specific businesses, such as the number of charity shops, off-licenses and betting shops are also available (LDC, 2017a).

Following LDC’s own methodology (LDC, 2018), our sample consists of the ‘leading’ towns and cities in Wales—namely those with 40 or more premises with retail or leisure use. As shopping centre and retail park remits are distinctly different to those of town centres, these have been removed from our sample (Dolega, et al., 2016).

### Developing Catchments for Town Centres in Wales

A town’s catchment area is defined as the group of locations from which regular visitors are drawn (Dolega et al., 2016). The literature identifies a number of approaches for catchment area delineation. The derivation of buffers around each centre using straight-line distances, service areas based on travel-distance or travel-time along a network, or more sophisticated techniques, such as the creation of thiessen polygons or location-allocation models, go some way towards the demarcation of catchment areas (Birkin et al., 2017). However, these techniques assume that each centre has a monopoly over the patron’s living within the catchment, doing little to account for the influence of a centre’s relative attractiveness on consumer behaviour (Dolega et al., 2016; Birkin et al., 2017). To account for these factors we build and apply a bespoke Spatial Interaction Model (SIM) to delineate catchment areas for each centre in our sample.

SIMs and gravity-based models predict the movement, or flow, of people between a demand point, such as their place of residence, and a supply point, such as a town centre (Dolega et al., 2016; Birkin et al., 2017). Production-constrained SIMs are used frequently for catchment area delineation at the store or retail centre level, and are a widely-applied tools to support the commercial sector location planning process, for example for new store revenue estimation (Wilson, 2010; Birkin et al., 2017; Newing, Clarke & Clarke, 2015). Such models are often disaggregated to account for variations in demand, supply and distance metrics, allowing them to be fine-tuned for a specific purpose (see Birkin et al., 2017; Dolega et al., 2016; Newing et al., 2020; Wilson, 2010 for a range of examples).

By using a SIM to model the flows of consumers between residential origins and the competing town centres in our study, we are able to delineate an indicative spatial extent over which each retail centre in our sample is able to draw custom, giving us an approximated centre catchment area. Crucially, the use of a SIM enables these catchments to overlap, recognising that there is not a finite point at which consumers stop patronising a given centre. Rather, each centre will experience a distance decay effect, with consumers living in proximity to that centre more likely to visit that centre as opposed to those centres that are less proximate and thus less accessible. However, consumers will also be influenced by centre attractiveness and underlying drivers of accessibility, such as travel time. The SIM enables us to account for these factors, estimating the probability that a consumer living in a given LSOA will visit each local centre, allocating consumers to the centre with the highest likelihood of patronage. Thus, we generate more realistic catchments than other more computationally straightforward approaches, allowing catchments to overlap during the modelling process, whilst also ensuring that this is avoided in our final catchments to simplify later analysis.

The SIM applied in this study takes the form:

$$S_{ij}^{k} = A_{i}^{k} O_{i}^{k} W_{j}^{\alpha } \exp^{{( - \beta^{k} C_{ij} )}} ,$$

where ${S}_{ij}^{k}$ is the flow of people between LSOA centroid *i* and town centre *j* for person type *k.* ${A}_{i}^{k}$ is a balancing factor which takes into account the competition of other town centres and ensures that all the population is allocated to a town centre. I.e.:

$${\overset{\boldsymbol\circ}{\mathbf a}}_jS_{ij}^k=O_i^k.$$

This is calculated as follows:

$$A_i^k=\frac1{{\overset{\boldsymbol\circ}{\mathbf a}}_jW_j^a\;\exp^{\left(-\mathrm b^{\mathrm k}{\mathrm C}_{\mathrm{ij}}\right)}},$$

${O}_{i}^{k}$ is the total population in LSOA *i* by person type *k*. ${W}_{j}^{\alpha }$ is the overall attractiveness of town centre *j* (centre size), adjusted by alpha (α) to account for the additional attractiveness associated with specific non-size based centre attributes. $\exp^{{( - \beta^{k} C_{ij} )}}$ is the distance-decay term, with ${C}_{ij}$ representing the travel time ‘cost’ of travel between LSOA *i* and town centre *j*, and ${\beta }^{k}$ representing the distance-decay parameter, adjusted for person type *k*.

The following sections briefly outline the key model building and calibration steps undertaken within this study.

#### Calculating and Applying the Geographic Distance Parameter

Consumers prefer to undertake routine and regular shopping tasks near to where they live or work. Therefore, the probability of them visiting a town declines with distance—an effect that must be accounted for by our SIM (Birkin & Clarke, 1991; WRO, 2007b).

A matrix of the cost of travelling between demand and supply points (taken as LSOA Population Weighted Centroids and DCLG retail centre centroids respectively) along Ordnance Survey’s (OS) Integrated Transport Network (ITN) (Birkin et al., 2017) was created using ESRI’s Network Analyst™ extension. The use of ITN ensured that cost was calculated along existing routes, accounting for natural barriers. The cost was defined using time rather than distance as this accounts for additional parameters such as road type, speed limits and average speed (Birkin, Clarke & Clarke, 2010; Dolega et al., 2016). We did not account for diurnal variation.

A consumer’s income, their access to private transport, or the rurality of their local area, may influence their ability and willingness to travel (Birkin et al. 2010; Dolega et al., 2016; Birkin et al., 2017). Indicators of social grade, car ownership rates and population density (as proxies for income, access to private transport and rurality) were used to generate an index to capture this. The distance-decay parameter (beta) within the SIM was disaggregated on an LSOA-by-LSOA basis using this index, capturing these neighbourhood level differences in consumer behaviour.

#### Calculating and Applying the Supply Parameter

Travel cost acts as less of a deterrent amongst centres perceived to be more attractive (Birkin & Clarke, 1991). Within our SIM, attractiveness is accounted for by the size of a centre in terms of number of premises (Birkin & Clarke, 1991; Birkin et al. 2010), drawn from the LDC data. To account for additional influences on attractiveness we use similar centre characteristics to those used in Dolega et al. (2016), such as the proportion of occupied premises, the retail mix and the proportion of leisure businesses. These three factors have been used to generate an index of ‘additional centre attractiveness’. When applied to the *W*_j_ term using the alpha parameter (additional non-size based centre attractiveness) smaller centres can be seen as relatively more attractive within the SIM if they have lower vacancy rates, a more diverse retail mix and greater leisure opportunities than comparable or larger centres.

The model was calibrated and adjusted iteratively. Given the lack of consumer flow data, optimisation was undertaken based on our expert understanding of town centres in Wales. For example, a ‘capital factor’ of one, chosen pragmatically rather than empirically, was added to Cardiff to give Wales’ capital more prominence; the raw attractiveness index and the raw distance-decay index values were rescaled to reduce their influence (Dolega et al., 2016; Birkin et al., 2017), and; the attractiveness indices for LSOAs within 0.5 km (2.5 min) walk (as the average shopping trip distance in the UK) were increased by the power of two, making smaller centres more attractive to their local populations (Zacharias, 2001; Dolega et al., 2016).

**Fig. 1:**
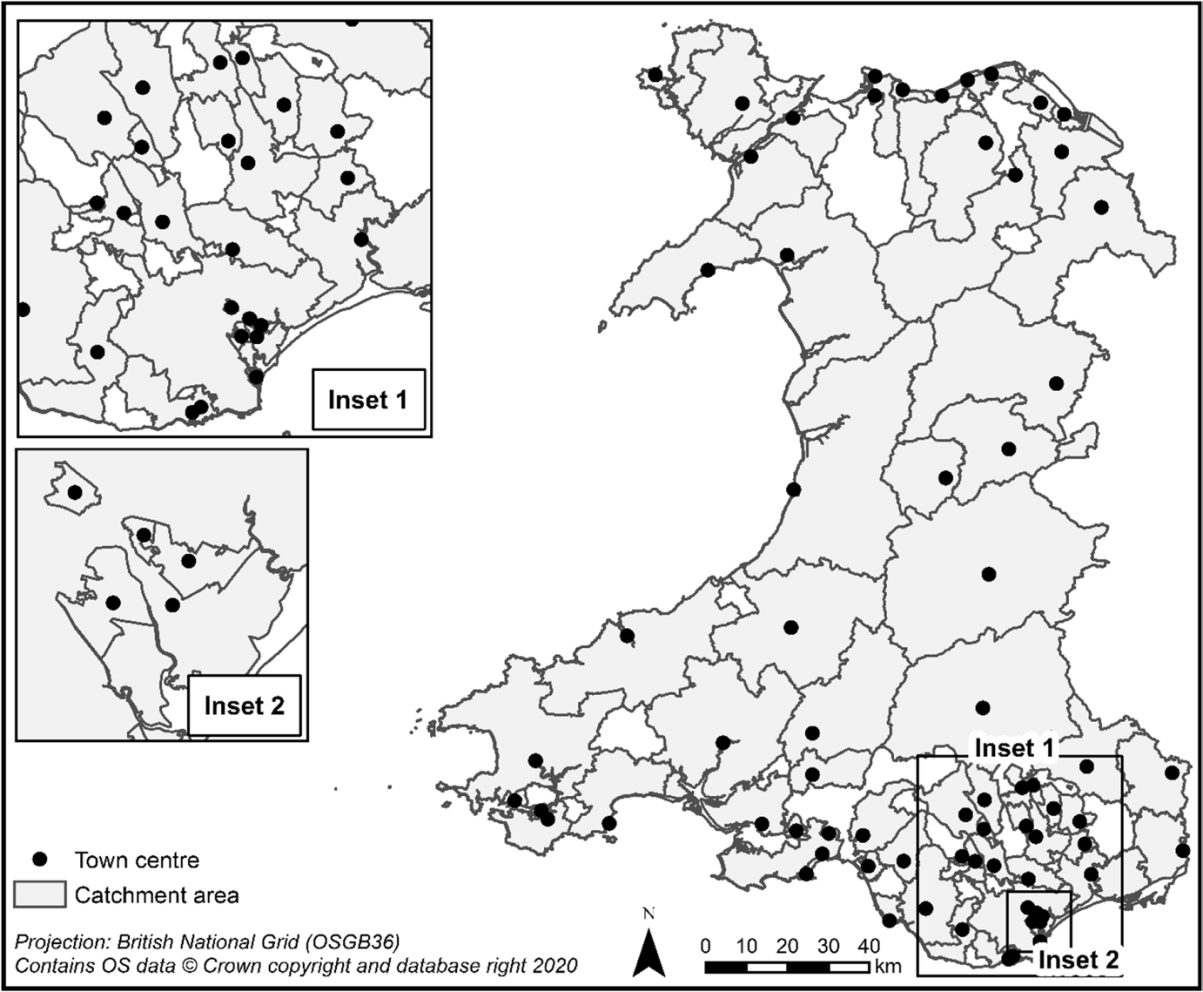
Final town centre catchment areas

The final catchment areas were generated for each town centre by combining LSOAs based on a predicated centre patronage of 50% or more (Dolega et al., 2016). This value, chosen pragmatically rather than empirically (Dolega et al., 2016), ensures that each LSOA is assigned to the single centre its population are most likely to patronise, simplifying later analysis. Small manual adjustments were made to six LSOAs which fell either just above or just below the threshold to make the catchments continuous (Fig. 1).

Crucially, these catchments were built via aggregation of LSOAs, enabling us to append additional small area characteristics to help better understand the catchment areas of towns in Wales. These are described in the next section.

#### Catchment Characteristics

We identified a number of demographic and socio-economic variables which would be beneficial in better understanding our delineated catchment areas (Table 1). LSOA level data from the Census 2011 were aggregated into the catchment area defined for each town centre.

**Table 1:** Catchment area metrics used to assess town centre performance by broad category

Demographic	Deprivation	Rurality	Accessibility
Age: 0–15 years	Households with no access to a car	Population density	Travel time to libraries: All transport
Age: 16–64 years	Unemployment	Proportion of urban communities	
Age: 65 + years	Proportion of communities in WIMD 1 and 2		
	Proportion of communities in WIMD 4 and 5		

We use the following metrics from the Welsh Index of Multiple Deprivation (WIMD) 2014. Since no direct indicators of town centre accessibility of available, and given that most libraries are based in the centre of towns, the ‘access to libraries’ metric acted as a proxy for accessibility to town centres. The proportion of a catchment’s LSOAs categorised amongst the most and least deprived in Wales, again using the WIMD 2014, was used as a measure of catchment deprivation, and the proportion of LSOAs categorised as Urban/Rural, taken from the ONS’ Rural–Urban Classification 2011, was used as a measure of a catchment’s rurality.

Next we move on to describe the development of our typology of town centres in Wales, which was ultimately used alongside these catchment characteristics explore differences in town centre performance.

### Developing a Typology of Towns in Wales

Our typology of town centres in Wales is created using k-means clustering, a partitional classification technique which groups individual cases into ‘clusters’ based on shared characteristics (Gale et al., 2016; Leventhal, 2016). This typology is based on supply side characteristics to match the requirements of PPW (Welsh Government, 2018), but it is then used later in conjunction with the demand characteristics, derived from the catchment areas delineated above, to better understand town centre performance. In this section we outline the variables selected and the process used to identify the most appropriate number of clusters. We then introduce the final six cluster typology.

#### Variable Identification

Firstly, we use the LDC dataset to identify town characteristics shown as key to understanding differences in town centre performance. To avoid data redundancy and multicollinearity the relationships between each variable were assessed using correlation coefficients. Variables exceeding correlations of ± 0.6 were examined in more detail and variables that identified similar characteristics were removed from the analysis (Gale et al., 2016). For example, when accounting for centre size, the DCLG area in metres^2^ was found to favour large sparsely populated towns and was therefore removed in favour of the total number of premises. Other variables, such as proportions of both independent and multiple retailers, were left in, despite strong correlations, as their inclusion added an important dimension to the typology.

To avoid our final typology being governed by centre size we followed the methodology outlined by the LDC (LDC, 2018), calculating: the proportion of Independent and Multiple retail premises out of the total retail premises; the proportion of Comparison, Convenience, Leisure and Services out of the total number of businesses; and, the proportion of charity shops, bookmakers, cheque cashers and off licenses as proportions of all premises. Variables were tested for normality, with skewed variables transformed using reflections and/or base-10 logarithms (Vickers, Rees & Birkin, 2005; Gale et al., 2016). As proportions of bookmakers, cheque cashers and off licenses (sellers of alcoholic drinks for consumption off the premises) were highly skewed, these were replaced with a single Alcohol, Money and Gambling (AMG) index. The final set of variables was then standardised using Z-scores and categorised according to broad type (Gale et al., 2016). Our final list of variables includes town size, retail mix and the prevalence of ‘weak’ retailers such as charity shops and those identified by the AMG index, which are often seen as undesirable fillers of vacant premises (Table 2) (Dolega et al., 2016).

**Table 2:** Town centre characteristics used to create the final typology

Retail mix	Weak retail mix	Size of centre
Independent	Charity shops	Total premises
Multiple	Alcohol, Money, Gambling (AMG)	
Comparison		
Convenience		
Leisure		
Service		

#### Identifying a Solution

The k-means process was run iteratively for several solutions containing different numbers of cluster groups. For reasons of brevity we have not provided a detailed discussion of the diagnostics used to identify the most appropriate solution. However, diagnostics included: (1) testing the distribution of town centres between cluster groups by calculating the range between the group with the highest and lowest counts for each solution; (2) avoiding groups with small counts by setting a minimum number of town centres per group per solution as half the mean average distribution of towns per group; (3) testing the homogeneity of each solution by calculating the average distance from the cluster centre, with more compact, homogenous clusters preferred; (4) setting a preference towards smaller numbers of cluster groups for ease of analysis and more effective visualisation (Vickers et al.,^2005^; Gale et al., 2016; Leventhal, 2016).

These diagnostics determined that a six cluster solution offered the most even distribution of town centres between clusters (a low range), whilst also avoiding clusters containing low counts of centres and maintaining homogeneity, minimising the variation within cluster groups and maximising variation between cluster groups. Clusters were named based on their dominant characteristics and pen portraits produced for each cluster. These pen portraits also draw upon the catchment characteristics derived above to better understand the typical communities each town type is situated in.

#### The Final Typology

**Fig. 2:**
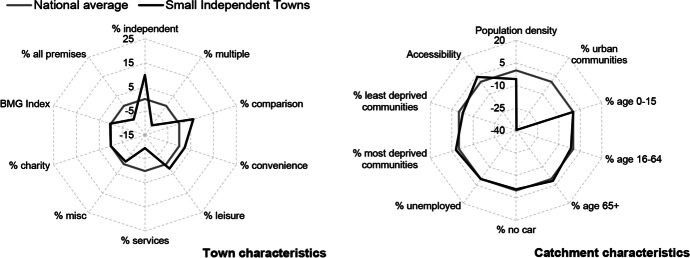
Town and catchment characteristics: Small Independent Towns

**Fig. 3:**
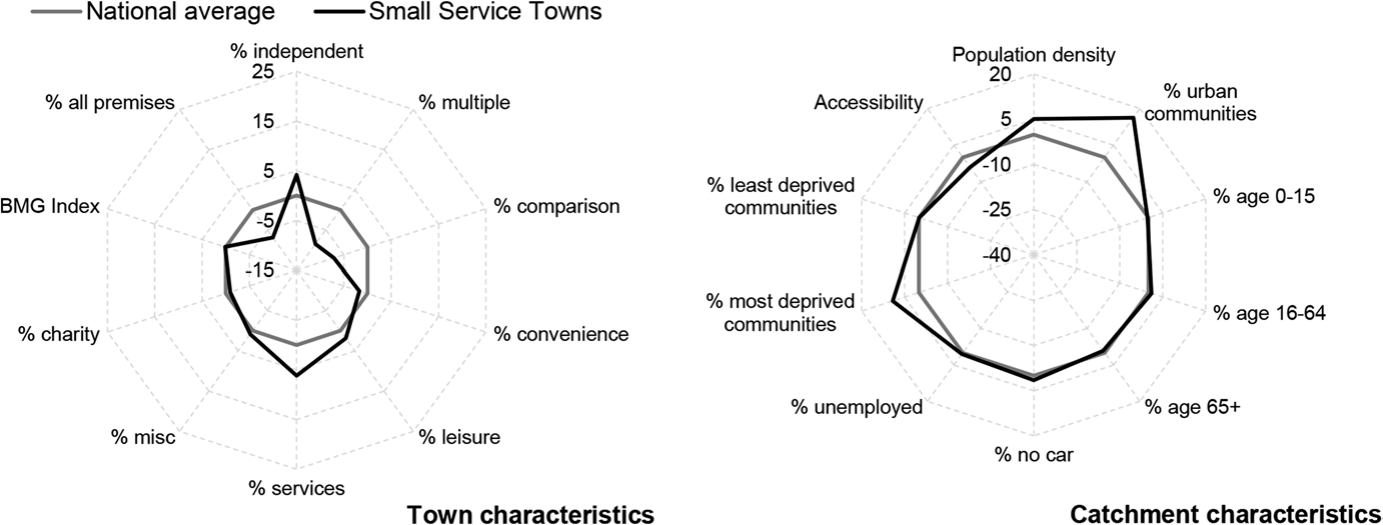
Town and catchment characteristics: Small Service Towns

**Fig. 4:**
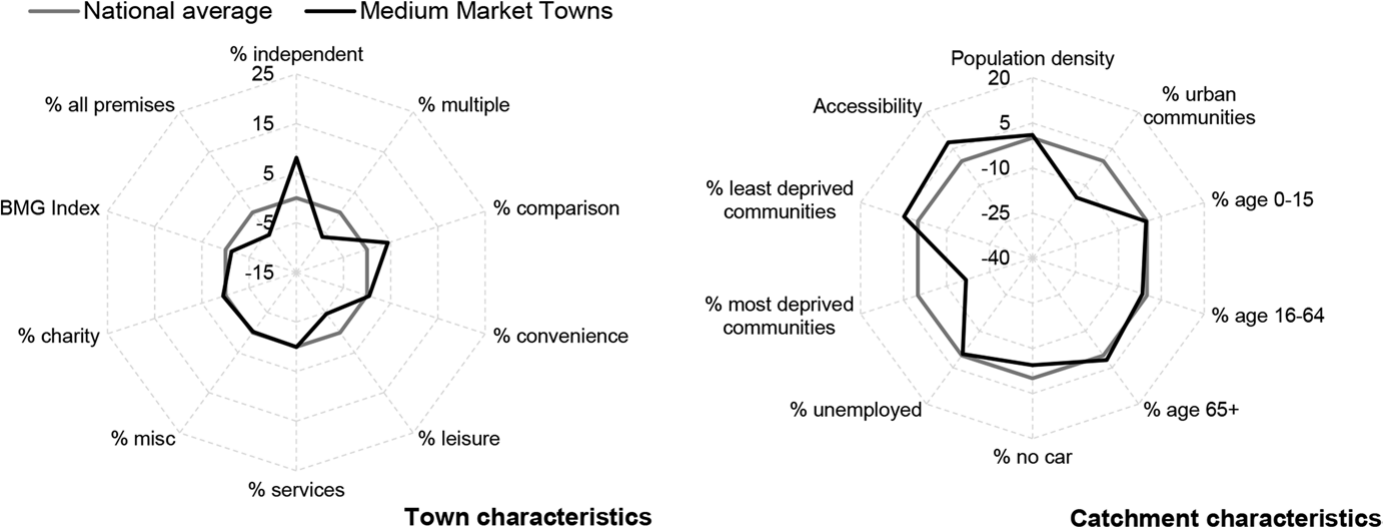
Town and catchment characteristics: Medium Market Towns

**Fig. 5:**
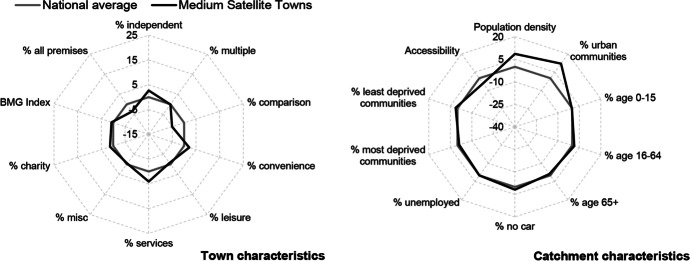
Town and catchment characteristics: Medium Satellite Towns

**Fig. 6:**
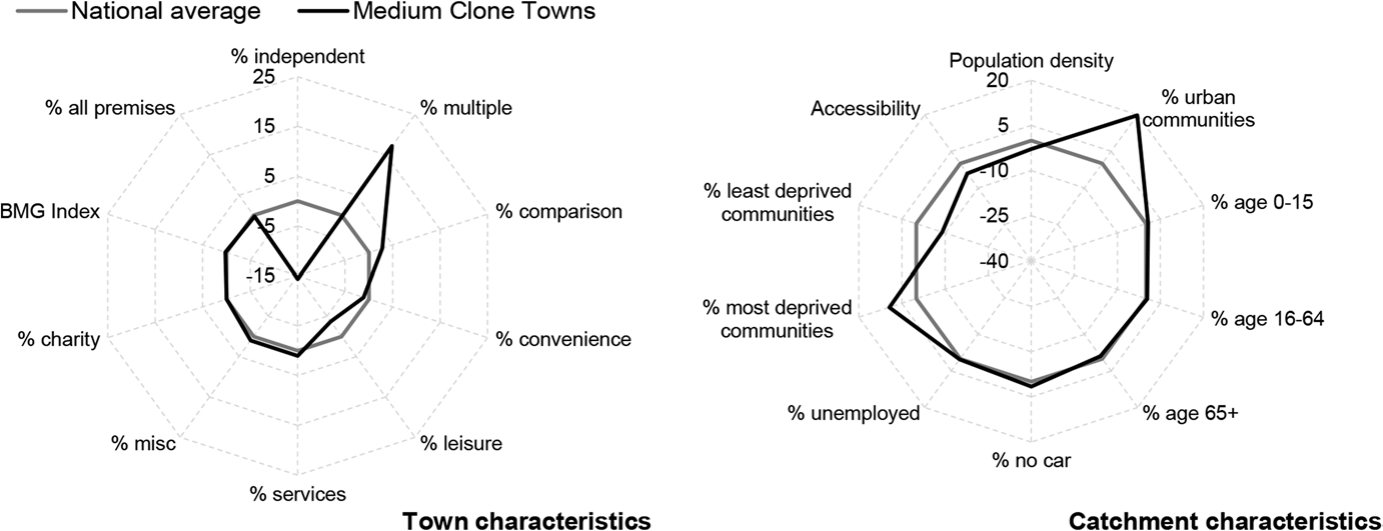
Town and catchment characteristics: Medium Clone Towns

**Fig. 7:**
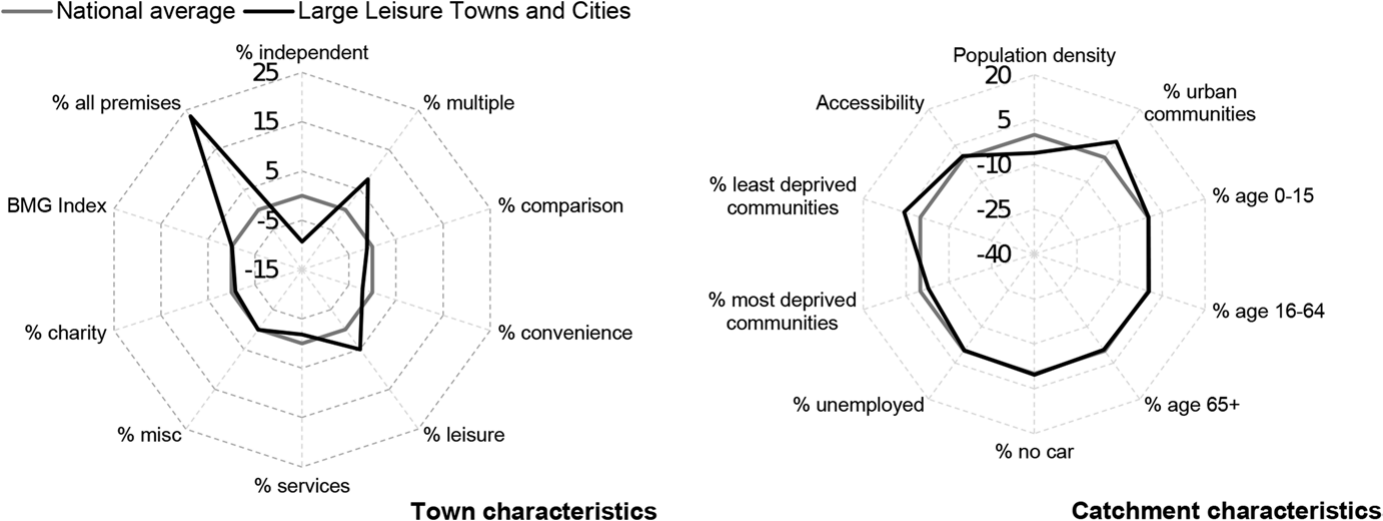
Town and catchment characteristics: Large Leisure Towns and Cities

**Fig. 8:**
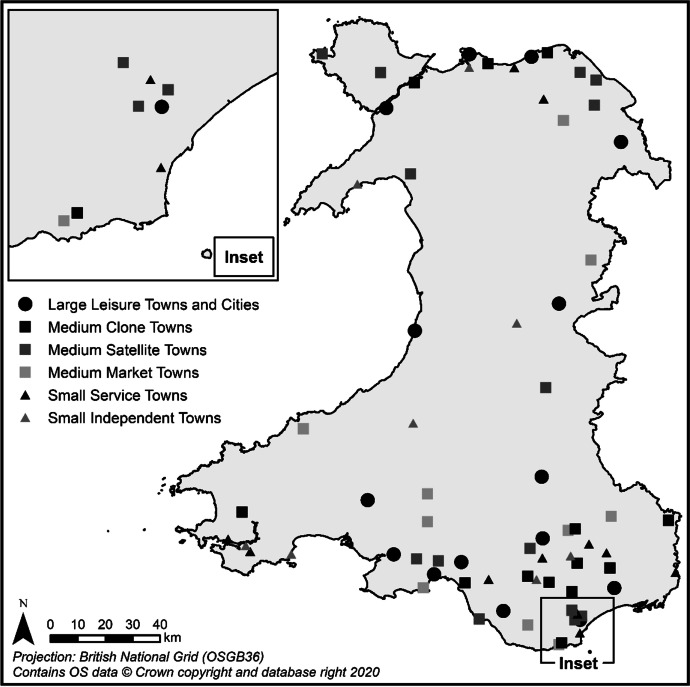
The final typology of town centres in Wales

Table 3 and Figs. 2, 3, 4, 5, 6 and 7 report summary and visual descriptions of towns typical to each cluster group. The final distribution of the typology can be seen in Fig. 8.

**Table 3:** Summary characteristics of the final typology

*Dominant characteristics*	*Small Independent Towns*	*Small Service Towns*
Size (mean premises per town)	Small (avg. 92 premises)	Small (avg. 93 premises)
Retail type	Independent	Independent
Business type	Comparison, convenience and leisure	Service and leisure
Catchment	Rural marginally deprived communities with relatively poor accessibility	Urban deprived communities with relatively good accessibility
Examples	Tenby and Conwy	Milford Haven and Pontypool
*Dominant characteristics*	*Medium Market Towns*	*Medium Satellite Towns*
Size (mean premises per town)	Medium (avg. 105 premises)	Medium (avg. 123 premises)
Retail type	Independent	Mix of independent and multiple
Business type	Comparison	Convenience and service
Catchment	Rural, affluent, retirement communities with relatively poor accessibility	Urban working age communities of mixed affluence and relatively good accessibility
Examples	Cowbridge and Abergavenny	Holyhead and the suburbs of cities (e.g. Whitchurch)
*Dominant characteristics*	*Medium Clone Towns*	*Large Leisure Towns and Cities*
Size (mean premises per town)	Medium (avg. 155 premises)	Large (avg. 390 premises)
Retail type	Multiple	Multiple
Business type	Comparison	Leisure
Catchment	Urban deprived communities with relatively good accessibility	Urban affluent communities
Examples	Bangor and Cwmbran	Wrexham, Aberystwyth and Cardiff

*Small Independent Towns* (Fig. 2) represents the smallest towns in the typology, with 92 premises on average. They are characterised by independent retailers and comparison, convenience and leisure businesses with low proportions of multiples and services. Catchment areas are predominantly rural, with relatively poor accessibility. *Small Service Towns* (Fig. 3) are also small, with an average of 93 premises per town. They consist of independent, service and leisure businesses, with low proportions of multiple, comparison and convenience businesses. They tend to be located in urban, deprived catchment areas with relatively good accessibility. *Medium Market Towns* (Fig. 4) have an average of 105 premises per town. Businesses tend to be independent retailers selling comparison goods amongst rural, affluent and retirement communities where accessibility is relatively poor. *Medium Satellite Towns* (Fig. 5) are slightly larger than Medium Market towns with 123 premises per town on average. Although there are marginally higher proportions of independents than multiples the mix is relatively even. Businesses are often focused on convenience goods and services as opposed to leisure and comparison goods. Catchments tend to be urban communities with marginally higher proportions of working age residents than the national average and relatively good accessibility. *Medium Clone Towns* (Fig. 6) are larger towns with an average of 155 premises per town. They are dominated by multiple retailers often selling comparison goods amongst urban, deprived communities with relatively good accessibility. *Large Leisure Towns and Cities* (Fig. 7) are the largest towns and cities in Wales, with an average of 390 premises per place. These tend to be characterised by multiple leisure businesses and tend to be found in urban affluent communities.

## Utilising Our Tool for Evaluating Town Centre Performance

The previous sections of this paper have described the creation of a typology of Welsh town centres which can be used, alongside the characteristics from our derived catchment areas, as a tool to better understand town centre performance. In this section we illustrate the application of this tool.

We begin by providing some context to the state of town centres in Wales between 2012 and 2016, a period contemporary to the data used in this study. Using these national averages as a benchmark we then consider the average performance of each of our town types. Following this, we will present two case studies of towns in the south Wales valleys. These are benchmarked against the average performance metrics of towns of the same type, exemplifying the use of this tool in better understanding differentials in town centre performance as an aid to decision makers.

Four measures of retail vacancy are used for town benchmarking purposes (Dolega et al., 2016; Dolega & Lord, 2020): (1) total retail vacancy rates in 2012; (2) total retail vacancy rates in 2016; (3) persistent vacancy, which measures the proportion of premises identified as being left vacant for 3 years or over in 2016, and; (4) churn, which measures the proportion of premises which have been vacant for less than a year in 2016.

### National Trends in Retail Vacancy from 2012–2016

LDC reports indicate that retail vacancy rate fell from 15.9 to 14.7% amongst towns and cities in Wales between 2012 and 2016, with towns accounting for the majority of this change (LDC, 2017b). The trend amongst cities is less clear, with rates fluctuating at around 22%. In 2016, churn accounted for 2.2% of premises, 2.9% in cities and 2.0% in towns. Persistently vacant premises accounted for 4.8% of premises, 6.1% in cities and 4.5% in towns. In terms of town centre composition the LDC suggests that proportions of comparison businesses were reducing over this period, whereas proportions of convenience and service businesses had seen a small increase (LDC, 2017b). Proportions of independent occupiers had also increased, suggesting a shift towards independent businesses in Wales (LDC, 2017b).

This evidence suggests that Wales’ towns are diversifying away from retail, a finding consistent with evidence from other parts of the UK (Findlay & Sparks, 2012; Wrigley & Lambiri, 2014; LDC, 2017b). A diversification of Wales’ towns and the reduction in retail vacancy rates over this period suggests that towns in Wales were, in general, getting healthier.

### Retail Vacancy by Town Type

**Fig. 9:**
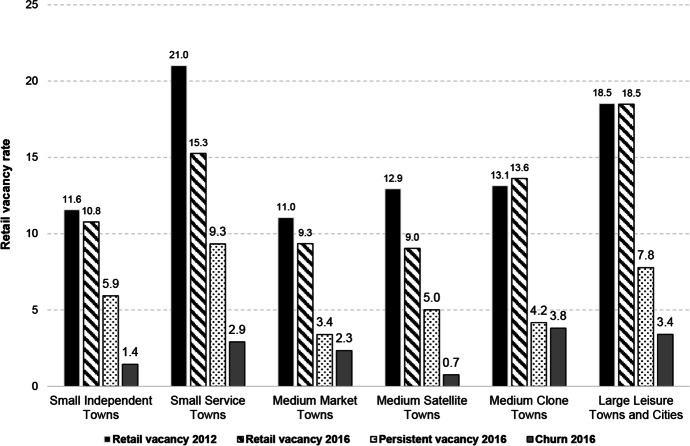
Change in performance by type of town (2012 – 2016)

We move on to explore town centre performance in Wales using our typology, linking trends in vacancy rate back to the key town and catchment characteristics outlined in the pen portraits (Figs. 2, 3, 4, 5, 6 and 7). Our results (Fig. 9) suggest that types of town with higher proportions of independent occupiers and higher business diversity, such as Medium Satellite Towns, Medium Market Towns and Small Independent Towns are associated with lower rates of retail vacancy in 2016, at 9.0%, 9.3% and 10.8% respectively. This is, perhaps, unsurprising: good quality independent retailers anchor a town centre by giving it distinctiveness and character, and recent consumer trends favour convenience shopping (WRO, 2007a, b; Wrigley & Dolega, 2011; Birkin et al., 2017). Persistent vacancy rates are lowest amongst Medium Market Towns (3.4%) and perhaps this is down to their affluent rural catchment areas, with higher spending power and less choice (WRO, 2007a, b; Wrigley & Dolega, 2011).

Retail vacancy amongst Small Service Towns, which was the highest of all town types in 2012 at 21%, had seen the largest reduction, dropping by 5.7 percentage points (pp) to 15.3% by 2016. These towns are often positioned in deprived urban communities, which should, perhaps, be associated with higher rates of vacancy. However, they are also characterised by high proportions of service businesses and previous research suggests that such services are key drivers of footfall as they often cannot easily be offered elsewhere (Coca-Stefaniak, 2013; Wrigley & Lambiri, 2014). Perhaps this reduction in vacancy shows the positive impact such services can have on town centre performance, particularly amongst less affluent communities. A large reduction in vacancy rates (3.9 pp) has also been seen amongst Medium Satellite Towns, another town type characterised by high proportions of services, which perhaps corroborates this trend. It should be noted, however, that persistent vacancy rates are still highest amongst Small Service Towns at 9.3%. This suggests that there is still a need to repurpose existing retail stock (Findlay & Sparks, 2010, 2012; Wrigley & Dolega, 2011) with a focus, perhaps, on service businesses.

Whilst the vacancy rate amongst Medium Clone Towns was still lower than the national average in 2016 at 13.6%, this is the only type to have experienced an increase in vacancy rates between 2012 and 2016 (0.5 pp). These towns are dominated by multiple and comparison goods businesses and perhaps this is a reflection of the fact that such towns often lack the personality and diversity to encourage visits by a broad range of consumers (Wrigley & Dolega, 2011). It is suggested that these types of towns may be the most vulnerable to future change.

Finally, vacancy rates in Large Leisure Towns and Cities are consistently high in 2012 and 2016 at 18.5% in both years. Whilst this may be surprising given the strong presence of leisure businesses and their association with contemporary trends (Grimsey, 2018), these static rates may be a facet of the amount of retail stock in larger places. This suggestion may be enforced by the relatively high levels of persistent vacancy (7.8%) and churn (3.4%) in these places and, similarly to Small Service Towns, may suggest a requirement to repurpose existing retail stock.

### Using the Tool to Benchmark Individual Towns

Having exemplified how our tool can be used to better understand differentials in town centre performance by broad category, we move on to showcase its application to individual towns by introducing two case study examples. These have been selected by using the typology to identify town centres which deviate notably in performance from the benchmark for their type; in itself, one key usage of our tool (Coca-Stefaniak, 2013; Wrigley & Dolega, 2011). These are considered, along with catchment characteristics, to identify reasons behind these deviations and to make suggestions for improvements, thus showing how it can be used to inform decision making.

#### Case Study 1: Cwmbran

**Fig.10:**
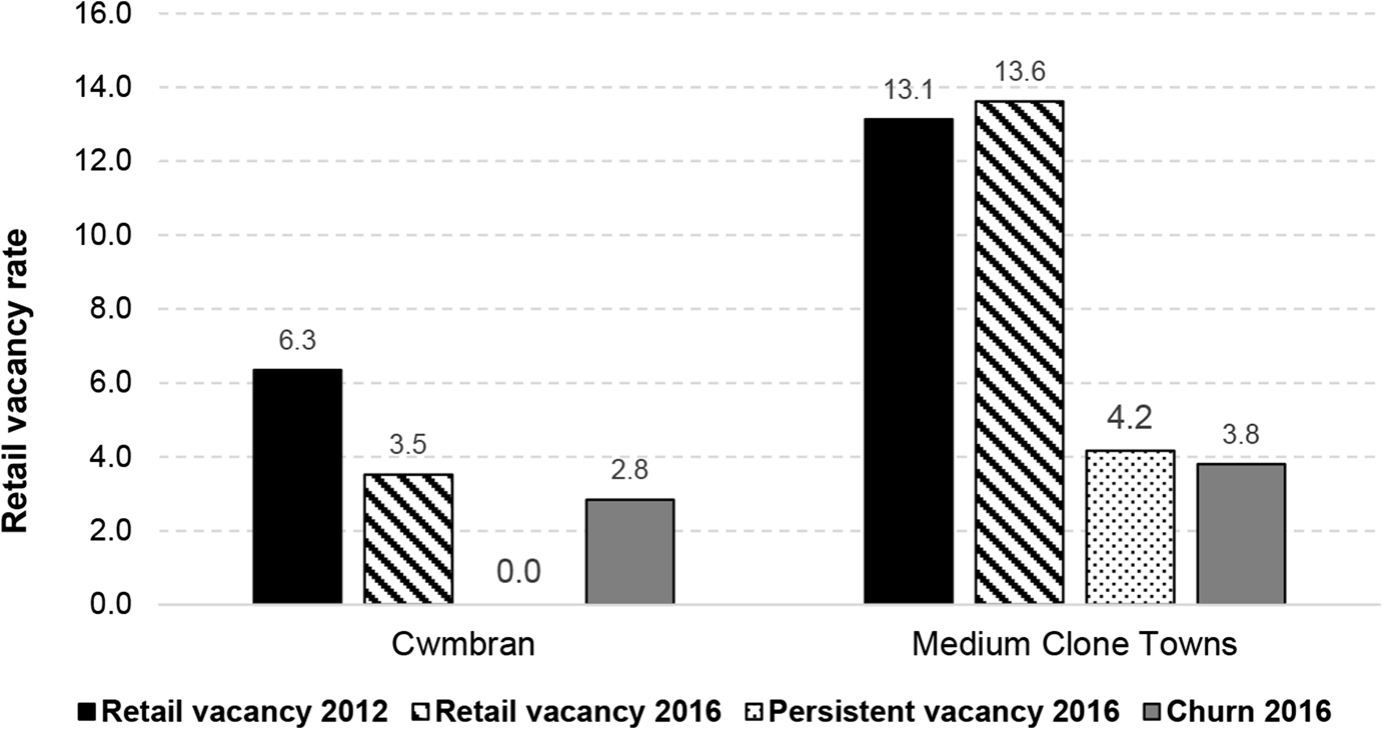
Benchmarking Cwmbran’s performance

**Fig. 11:**
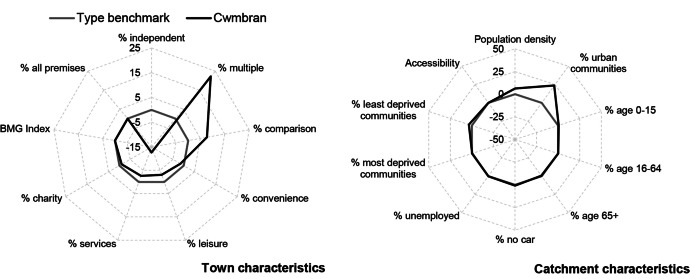
Benchmarking Cwmbran’s characteristics

Cwmbran, a town in Torfaen Local Authority in the south Wales valleys, has been classified as a Medium Clone Town according to its retail mix. Comparing this town against the benchmark shows that Cwmbran is performing relatively well compared to its peers, with vacancy rates in 2016 over 10 pp lower than the benchmark (Fig. 10). A closer examination of Cwmbran’s catchment area indicates that it is located amongst higher proportions of urban communities and marginally higher proportions of affluent communities than other towns of this type (Fig. 11). Its town centre characteristics suggest notably higher proportions of multiples and comparison retailers when compared to its peers, with lower than average proportions of independents, convenience, leisure and services.

Although Cwmbran is located in a marginally less deprived catchment, trends discussed above suggest that Cwmbran’s urban character and poor retail mix should indicate poor performance. However, this town centre, developed as a result of the New Town Act 1946 (UWP, 2021) is known to consist of a purpose-built shopping centre, with pedestrianisation, parking and high-quality public amenities, perhaps making it more attractive to consumers and investors (TCBC, 2007, 2015). Reports also show that Cwmbran has seen careful town centre management and significant investment and regeneration on several occasions since 2003, including in 2008, 2011 and 2017 (TCBC, 2007; BBC, 2009; SWA, 2017). This suggests that effective town centre management and regular regeneration, as well as a high quality physical environment, plays an important part in attracting consumers and maintaining performance (Powe, 2012; Wrigley & Lambiri, 2014).

Medium Clone Towns have, however, been identified as amongst the most vulnerable to change. Therefore, we would recommend that Cwmbran diversifies its usage—trying to establish a greater presence of services, leisure, independents and USPs. Town centre managers are known to be doing this by developing a cinema, bowling alley and trying to make the most of the Brecon and Monmouthshire Canal (TCBC, 2004, 2007).

#### Case Study 2: Tredegar

**Fig. 12:**
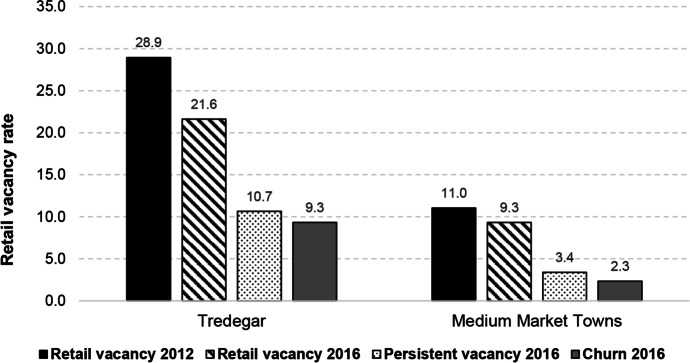
Benchmarking Tredegar’s performance

**Fig. 13:**
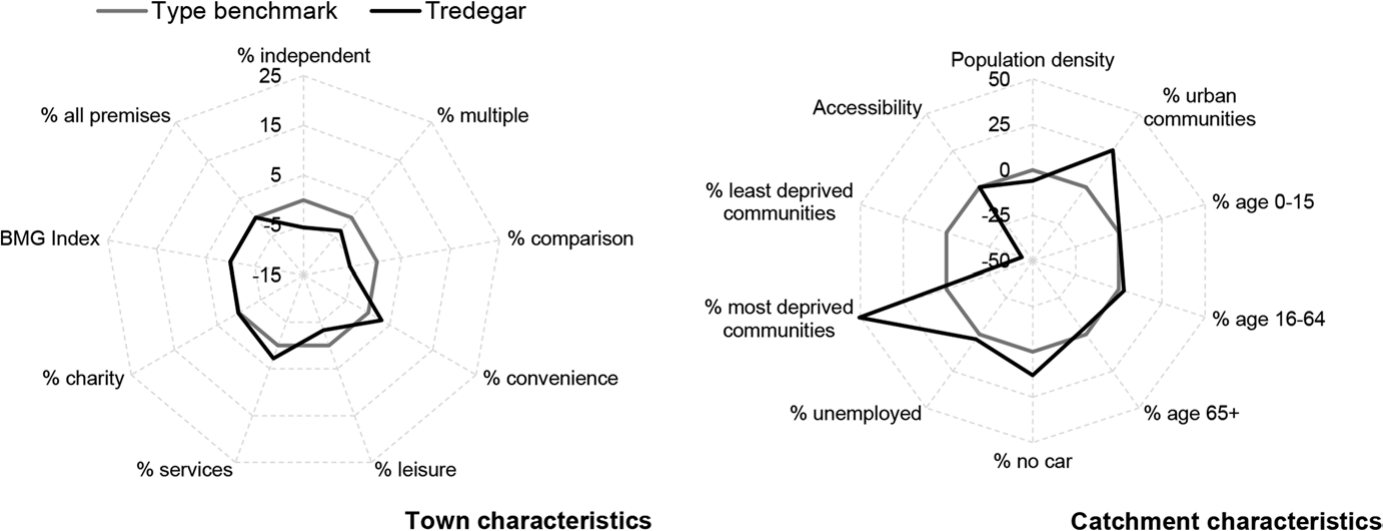
Benchmarking Tredegar’s characteristics

Tredegar, a town in Blaenau Gwent Local Authority in the south Wales valleys, has been classified as a Medium Market Town based on its retail mix. Our tool indicates that it is performing poorly compared to its peers, with vacancy rates in 2016 around 12 pp higher than the category benchmark (Fig. 12). A closer examination of its catchment area suggests that Tredegar is situated in more urban, deprived communities than other towns of this type (Fig. 13). A Medium Market Town’s diverse mix of businesses, focused on comparison goods and independents, is normally associated with more affluent, rural catchments and is, perhaps, not appropriate for a town centre such as Tredegar. Whilst independent businesses can be used to anchor a town, their lack of resilience can leave poorly performing towns fragile (Wrigley & Dolega, 2011). Given the correlation between service businesses and improvements in town centre performance in deprived areas, we recommend that Tredegar’s stakeholders focus on attracting more service businesses to the town (Coca-Stefaniak, 2013; Wrigley & Lambiri, 2014).

## Discussion

Through the novel application of Welsh Government planning policy this research has created a new tool for understanding and evaluating Wales’ leading towns and cities. This tool is based on both a national typology of town centre characteristics and the catchment area characteristics of the local residential population. In applying the tool, our goal was to benchmark towns of similar types, offering suggestions for differentials in town centre performance. Ultimately we sought to provide the robust data-driven evidence highlighted by PPW as important for supporting strategies intended to improve town centre performance (Paragraph 4.3.6, Welsh Government, 2018). We identify key differences in town centre performance based on retail vacancy rates (Dolega & Lord, 2020) and, as a result, we have raised several suggestions for the consideration of stakeholders.

Firstly, our findings suggest that towns with higher proportions of independent occupiers and higher business diversity are often associated with lower vacancy rates (WRO, 2007a, b; Wrigley & Dolega, 2011; Birkin et al., 2017), suggesting that stakeholders should consider increasing the vitality of towns, with a particular focus on independent retailers. Secondly, we recommend that stakeholders responsible for significantly underperforming towns concentrate on service businesses (Coca-Stefaniak, 2013; Wrigley & Lambiri, 2014), repurposing and advertising retail stock accordingly. This appears to have reduced vacancy rates, even in towns situated in less affluent communities. However, such services should be chosen carefully to avoid businesses which could be potentially harmful to a community, such as betting shops (Coca-Stefaniak, 2013; Wrigley & Lambiri, 2014). Thirdly, we have identified that places classified as Large Leisure Towns and Cities and Small Service Towns often have high proportions of persistently vacant premises. This suggests that there is a need to consider repurposing existing retail stock in these places, perhaps with more of a focus on service businesses (Findlay & Sparks, 2010, 2012; Wrigley & Dolega, 2011). Finally, we have identified Medium Clone Towns as the town type most vulnerable to change and suggest stakeholders attempt to increase the diversity of these towns by attracting more independent retailers and convenience, service and leisure businesses (Wrigley & Dolega, 2011; Birkin et al., 2017).

In addition to using our tool to better understand differentials in performance by broad category, we also exemplified its application to individual towns through two case study examples. In our first case study, we showed Cwmbran, a Medium Clone Town, to be outperforming peers in the same category, suggesting town centre management and regeneration strategies as a key reason for this (Wrigley & Lambiri, 2014). However, given the potential weaknesses of Medium Clone Towns, we were also able to recommend that stakeholders for Cwmbran look to diversify its business mix towards leisure and services. In our second case study, we showed that Tredegar, a Medium Market Town, appears to be underperforming compared to its peers. Our analysis shows that Medium Market Towns tend to be situated in affluent rural communities, whereas Tredegar is situated in an urban deprived community. Based on this finding, we recommend that Tredegar focuses more on services given their link with better performing towns in urban and deprived areas (Coca-Stefaniak, 2013; Wrigley & Lambiri, 2014). We have used such examples to showcase how stakeholders could apply this tool to benchmark towns, and envisage its application ultimately helping them develop and apply interventions appropriate to towns under their own jurisdiction.

## Conclusion

Planning Policy Wales highlights the importance of towns as places in contemporary Wales. Reversing declines in their usage requires a better understanding of the impact consumer trends and global events have on town centres at a local level. Since we have undertaken this research, Wales and the rest of the UK has experienced the COVID19 pandemic. This has had a disproportionate impact on the high street due to social distancing, stay at home orders and the closure of many retail and leisure premises during periods of lockdown. The short- and long-term impacts of the pandemic on the retail landscape are not yet clear, but town and city centres will undoubtedly need investment and targeted interventions to help them recover. Our tool, presented here, provides both a starting point and the robust evidence needed to support decision making, offering insight into the relationships between a town, its catchment and other towns and catchments with similar characteristics. Linked to up-to-date data on town centre metrics such as vacancies, the differential impact of the pandemic on town centres across Wales can be evaluated in a consistent and robust manner.

A number of limitations and opportunities for future work have been identified as a result of this research. Firstly, our data and town centre boundaries are slightly dated and so future work would involve updating our typology using more contemporary data on town centre characteristics and more up-to-date boundaries (Pavlis et al., 2018). Additionally, should the data become available, would also envisage: broadening the sample to more than just the 71 towns and cities in Wales used here; broadening the data used to incorporate a wider range of cultural and environmental metrics and; broadening the metrics used to measure town centre performance, including the likes of footfall. This would provide us with a more complete picture of both town centre types and town centre performance in Wales. In terms of alterations to our methodology, we have developed this tool as two separate components (town characteristics and catchment characteristics), aligning it with PPW. However, there may be some benefit in combining these into a single typology consisting of both town and catchment characteristics in Wales, similar to Dolega et al. (2019)’s work for GB.

## Data Availability

Not applicable.
